# Applying AI to Safely and Effectively Scale Care to Address Chronic MSK Conditions

**DOI:** 10.3390/jcm13154366

**Published:** 2024-07-26

**Authors:** Anabela C. Areias, Dora Janela, Robert G. Moulder, Maria Molinos, Virgílio Bento, Carolina Moreira, Vijay Yanamadala, Fernando Dias Correia, Fabíola Costa

**Affiliations:** 1 Sword Health, Inc., Draper, UT 84043, USA; a.areias@swordhealth.com (A.C.A.); d.janela@swordhealth.com (D.J.); robertgm111@gmail.com (R.G.M.); mmolinos@swordhealth.com (M.M.); vbento@swordhealth.com (V.B.); c.moreira@swordhealth.com (C.M.); v.yanamadala@swordhealth.com (V.Y.); fcorreia@swordhealth.com (F.D.C.); 2 Institute for Cognitive Science, University of Colorado Boulder, Boulder, CO 80309, USA; 3 Instituto de Ciências Biomédicas Abel Salazar, 4050-313 Porto, Portugal; 4 Department of Surgery, Quinnipiac University Frank H. Netter School of Medicine, Hamden, CT 06473, USA; 5 Department of Neurosurgery, Hartford Healthcare Medical Group, Westport, CT 06103, USA; 6 Neurology Department, Centro Hospitalar e Universitário do Porto, 4099-001 Porto, Portugal

**Keywords:** musculoskeletal pain, physical therapy, telerehabilitation, eHealth, artificial intelligence, workflow, clinical decision support

## Abstract

**Background/Objectives**: The rising prevalence of musculoskeletal (MSK) conditions has not been balanced by a sufficient increase in healthcare providers. Scalability challenges are being addressed through the use of artificial intelligence (AI) in some healthcare sectors, with this showing potential to also improve MSK care. Digital care programs (DCP) generate automatically collected data, thus making them ideal candidates for AI implementation into workflows, with the potential to unlock care scalability. In this study, we aimed to assess the impact of scaling care through AI in patient outcomes, engagement, satisfaction, and adverse events. **Methods**: Post hoc analysis of a prospective, pre-post cohort study assessing the impact on outcomes after a 2.3-fold increase in PT-to-patient ratio, supported by the implementation of a machine learning-based tool to assist physical therapists (PTs) in patient care management. The intervention group (IG) consisted of a DCP supported by an AI tool, while the comparison group (CG) consisted of the DCP alone. The primary outcome concerned the pain response rate (reaching a minimal clinically important change of 30%). Other outcomes included mental health, program engagement, satisfaction, and the adverse event rate. **Results**: Similar improvements in pain response were observed, regardless of the group (response rate: 64% vs. 63%; *p* = 0.399). Equivalent recoveries were also reported in mental health outcomes, specifically in anxiety (*p* = 0.928) and depression (*p* = 0.187). Higher completion rates were observed in the IG (79.9% (N = 19,252) vs. CG 70.1% (N = 8489); *p* < 0.001). Patient engagement remained consistent in both groups, as well as high satisfaction (IG: 8.76/10, SD 1.75 vs. CG: 8.60/10, SD 1.76; *p* = 0.021). Intervention-related adverse events were rare and even across groups (IG: 0.58% and CG 0.69%; *p* = 0.231). **Conclusions**: The study underscores the potential of scaling MSK care that is supported by AI without compromising patient outcomes, despite the increase in PT-to-patient ratios.

## 1. Introduction

Musculoskeletal (MSK) pain affects approximately 1.71 billion people worldwide [1], imposing a substantial societal and economic burden, which is reflected by USD 380.9 billion of yearly medical expenditure in the United States alone [2].

The urge to improve patient access to care has precipitated a transformative shift in MSK management towards digital rehabilitation [3,4] as a way to address geographical barriers [5,6]. However, beyond the unbalanced geographical distribution of healthcare providers, there is also an insufficient number of trained clinicians to meet the rising demand caused by a continuous increase in the prevalence of MSK conditions [1,7]. This has resulted in persistent access barriers and delayed care, resulting in costly care escalations, as well as a significant prevalence of clinician burnout in recent years [8]. These factors demand a new wave of innovation that can address care access issues in a broad, scalable, and sustainable manner.

In this setting, artificial intelligence (AI) has emerged as a powerful tool in healthcare [9]. Examples include the use of AI assistants to significantly reduce the documentation burden and physician burnout [8], and inpatient hospital surveillance systems to assist nurses by providing automatic alarms for patient monitoring [10].

Digital care programs (DCP) benefit from the automatic and centralized collection of data, making them well-suited for the integration of AI. AI systems can help scale MSK care by supporting monitoring and decision-making with data-driven insights, thereby reducing the workload and optimizing outcomes and efficiency, and reaching a higher number of patients in a sustainable way.

Research on AI tools in the context of MSK rehabilitation is still in its early stages. Current studies focus on initial stages of patient treatment, including the triaging [11] and phenotyping of patients [12], and predicting prognosis [13,14]. So far, however, research has not been focused on the impact of the introduction of scalability approaches in clinical outcomes. With this in mind, in this study, we aimed to evaluate whether AI-assisted care scalability compromises care delivery or if patients benefit similarly from high-quality care. To this end, we compared the clinical outcomes, as well as the engagement, satisfaction, and adverse events, of two cohorts with different physical therapists (PT)-to-patient ratios, whereby the cohort with the highest ratio was supported by a machine learning-based tool to enhance workflows and decision-making on patient progression. We hypothesized that patient outcomes would not be significantly different between cohorts, given the potentially optimized workflows obtained by the AI tool’s introduction on the increased-ratio cohort.

## 2. Materials and Methods

### 2.1. Study Design

This is a post hoc analysis of two prospective, single-arm, IRB-approved studies (New England IRB: 120190313 and Advarra IRB: Pro00063337), which were prospectively registered on ClinicalTrials.gov (NCT04092946, NCT05417685) on 17 September 2019 and 14 June 2022, respectively.

Both cohorts were recruited with the intent to investigate the clinical and engagement-related outcomes of patients with MSK undergoing a DCP. The comparison cohort (PT-to-patient ratio: 1:57 (SD 36), with no assisting AI tool) had patients enrolled between 18 June 2020 and 13 June 2022, whereas the intervention cohort (PT-to-patient ratio: 1:129 (SD 27), with AI tool assistance) enrollment period occurred from 21 November 2022 to 8 July 2023. A washout period was considered, to avoid hybrid situations with pilot testing (between 14 June 2022 and 20 November 2022).

### 2.2. Population

The beneficiaries of employer health plans who reported chronic musculoskeletal (MSK) pain (defined as persistent or recurrent pain lasting ≥ 12 weeks) in the ankle, elbow, hip, knee, low back, neck, shoulder, wrist, or hand were invited to participate in the study through a dedicated enrollment website.

Exclusion criteria included: (1) health conditions that were incompatible with at least 20 min of light to moderate exercise; (2) ongoing cancer treatment; and (3) the presence of signs or symptoms indicative of serious pathology (e.g., rapid progressive motor weakness, sensory alterations, or bowel or bladder dysfunction). All participants provided informed consent.

### 2.3. Intervention

#### 2.3.1. Digital Care Program

Both groups received a DCP (Sword Health Inc., Draper, UT, USA) consisting of exercise, education, and cognitive behavioral therapy (CBT) (as described elsewhere [15,16,17]), lasting up to 12 weeks according to each patient’s condition.

During onboarding, patients chose a PT who would be responsible for prescribing, monitoring, and adjusting the program according to the patient’s condition and goals, providing continuous support throughout the program. All PTs included in the study had a Doctor of Physical Therapy degree and a minimum of 3 years of experience.

Patients performed the exercise sessions independently at their convenience, using an FDA-listed class II medical device (Sword Health Inc., Draper, UT, USA) consisting of a dedicated tablet (software version 1.0 in the comparison group and 2.4 in the intervention group), motion tracking, a mobile app, and a cloud-based portal (Supplementary Figure S1). The exercise sessions were displayed on the tablet with real-time video and audio biofeedback on the patient’s execution. Data related to sessions, including exercise performance (correct and incorrect movements), range of motion, the number of sets and repetitions completed, and self-reported pain and fatigue levels felt during exercise were automatically collected and stored in the cloud-based portal. This information was accessed by the assigned PT, enabling remote and asynchronous monitoring and adjustments to the prescription whenever needed.

Tailored education and CBT components were delivered through a smartphone app in the form of written articles, videos, audio content, and interactive modules, as described elsewhere [15]. Bidirectional communication was ensured through a built-in secure chat system within the smartphone app and video calls.

#### 2.3.2. Workflow-Related AI Tool

**Figure 1:**
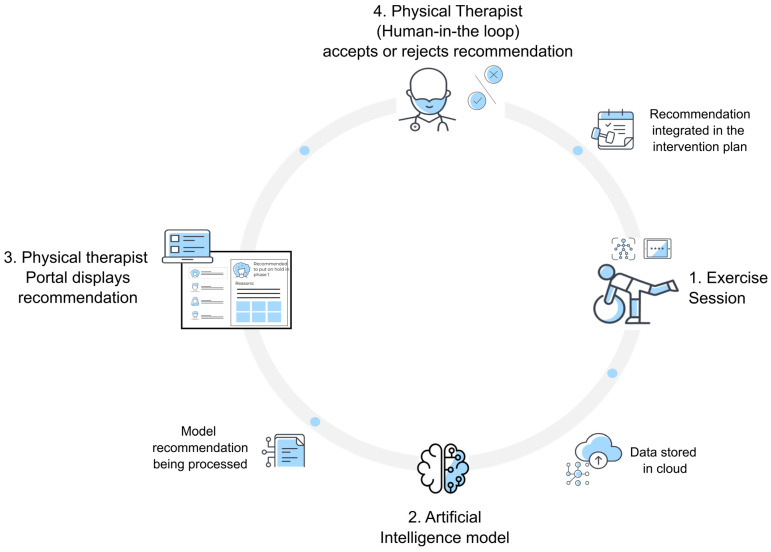
Schematic diagram of the AI tool. (1) Automatically collected data of a patient’s session are stored in the cloud and fed into an artificial intelligence (AI) model. (2) The AI model, primarily trained with previous physical therapist (PT) decisions, generates a recommendation about the possible adjustments to the patient’s care program (example: introduction of new exercises, increasing exercise dosage). (3) The recommendation is then displayed on the PT portal, along with the reasons behind the suggestions, enabling a more efficient revision of the patient’s clinical status to support the PT’s final decision. (4) The PT analyzes the recommendation, accepting or rejecting it, to support the final decision. Finally, a re-tailored intervention plan is sent to the patient’s tablet with a new updated session. This loop framework allows for streamlining adjustments to care programs to facilitate PT workflow.

Tasks involving data monitoring to stratify patients based on their need for clinical attention and program adjustments were deemed to be time-consuming by PTs and were, therefore, prioritized for a machine learning (ML)-based system. Subsequently, a multidisciplinary team combining data engineers, computer scientists, and PTs followed a human-in-the-loop framework [18] to develop a ML-based tool focused on streamlining adjustments to care programs (e.g., the introduction of new exercises or increasing exercise dosage). This tool provides recommendations to the PTs, as well as giving the reasons behind the suggestions, through the PT portal, enabling a more efficient revision of the patients’ clinical status and supporting the PT’s final decision (Figure 1 and Supplementary Table S1). The tool was primarily trained using previous PT decisions and fed with automatically collected data during each session (e.g., exercise performance and self-reported pain and fatigue during sessions; a detailed description of the input data can be found in Supplementary Table S2). Additionally, through a dynamic/iterative learning loop, the algorithm adapted and refined the recommendations, consequently improving its performance.

Algorithm performance was routinely tested against PTs’ manual decisions. In instances where the AI tool could not provide a recommendation (insufficient accuracy), a notification prompted the PT to resort directly to a manual decision.

The AI tool was deployed in sequential pilot tests (between 14 June 2022 and 20 November 2022) before broad deployment that reflected the increase in the PT-to-patient ratio.

#### 2.3.3. Comparison Group (CG)

This cohort benefited from the standard DCP described above, wherein the information stored in the portal was manually managed and prioritized by the PT, and where the PT-to-patient ratio was 1:57 (SD 36), which is in line with the 1:60 ratio for in-person therapies [19].

#### 2.3.4. Intervention Group: PT Portal Powered by AI Tool

The AI-intervention group (IG) received the same standard DCP as previously described, with a PT-to-patient ratio of 1:129 (SD 27), wherein the PTs were assisted by an AI tool integrated into the web portal.

### 2.4. Outcomes

Patient assessment was performed at baseline and at the 9th, 18th, and 27th sessions (according to the discharge time point) to analyze longitudinal changes between the baseline and program-end results.

PT-to-patient ratios were calculated month over month by averaging the number of patients being supported by the number of PTs available for a given month, regardless of the patient treatment stage.

Outcome measures comprised:

(1) Pain intensity, as measured by the Numerical Pain Rating Scale (NPRS) that is specific for the symptomatic body region: “Please rate your average pain over the last 7 days, from 0 (no pain at all) to 10 (worst pain imaginable)”. The response rate was calculated considering a minimum clinically important change (MCIC) of 30%. This threshold was defined based on the recommendations from the IMMPACT guidelines for clinical trials assessing chronic pain interventions [20];

(2) Mental health, with anxiety assessed by the 7-item Generalized Anxiety Disorder (GAD-7) scale (range 0–21) [21,22], and depression being assessed by the 9-item Patient Health Questionnaire (PHQ-9) (range 0–27) [22,23]. Higher scores denote worse symptomatology in both scales;

(3) Safety, which is assessed by the adverse event rate;

(4) Engagement, which is assessed by completion of the program (completion rate), average sessions per week, and communication between the PT and the patient;

(5) Satisfaction, which is assessed through the question “On a scale from 0 to 10, how likely is it that you would recommend this intervention to a friend or neighbor?”.

### 2.5. Safety and Adverse Events

Routine internal quality checks of the AI tool were conducted by comparing the algorithm’s recommendations against the manual decisions made by PTs to continuously assess its performance.

Patients were advised to report any adverse events (e.g., worsening of symptomatology, new symptoms, or other events that could interfere with the patient’s condition or the execution of the program) to the dedicated PT through the available communication channels for further assessment. These reports were documented and addressed using an internal software system, in accordance with internal quality and safety protocols. Additionally, the patient’s pain and fatigue levels during the exercise sessions (assessed by NRS; range 0–10) were collected at the end of each session.

### 2.6. Sample Size

The sample size estimation was based on the primary outcome—pain level—wherein a MCIC of 30% was selected, based on the psychometric properties of the scale [24]. Considering a power of 80%, a one-sided 0.05 significance-level non-inferiority study, and a 20% dropout rate [15,25], 2702 patients (1351 per group) would be necessary to detect a difference between the two groups.

### 2.7. Statistical Analysis

Descriptives were used to depict the patients’ demographic and clinical characteristics at baseline, along with engagement metrics. Comparisons between groups were performed using an independent samples *t*-test or the Mann–Whitney U test for continuous variables (with Bonferroni correction), or a chi-squared test for categorical variables. Both cohort reassessments were pivoted to 9, 18, and 27 sessions to ensure standardization of the reassessment timeframe.

Inverse probability weighting (IPW) was applied to control for confounding effects between groups [26] by differentially weighting each participant’s baseline characteristics using demographics that have previously been found to be meaningfully different: age, body mass index (BMI), and employment status. These weights were included in the data analysis to strengthen causal inference and were calculated for the whole cohort or for those with clinically relevant baseline scores (i.e., ≥5, to further compute any changes in the GAD-7 and PHQ-9 outcomes). Multiple-group latent growth curve analysis (LGCA) was used to estimate the overall changes for each outcome, based on trajectories across time, and to perform comparisons between groups following an intention-to-treat analysis. LGCA, which applies a structural equation model, recognizes correlations between repeated measures for the same individual, provides model fit measures, and incorporates full information maximum likelihood (FIML) to handle missing data [27]. FIML uses all available data at each time point from all participants to calculate the maximum likelihood estimates, outperforming multiple imputation by chained equations or listwise deletion [28]. The model was adjusted for each individual discharge time point. Cohen’s d effect sizes were calculated for all clinical outcomes by comparing changes between the groups, considering the following thresholds: 0.2—small, 0.5—medium, 0.8—large, and 1.3—very large [29].

The odds of being a responder for pain was calculated using logistic regression analysis, considering an MCIC of 30% [20].

A robust sandwich estimator was used in all models for standard errors. All statistical analyses were conducted using R (version 4.2.2; R Foundation for Statistical Computing) and Python (version 3.11.4, Python Software Foundation, Wilmington, DE, USA), and the level of significance was set at *p* < 0.05 for all tests.

## 3. Results

**Figure 2:**
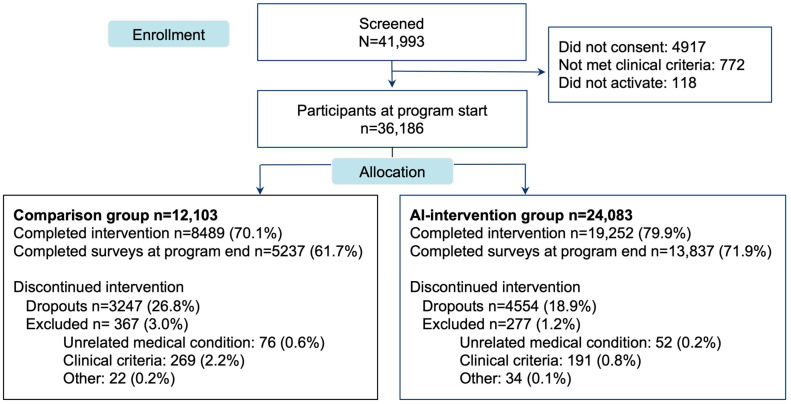
Study flow chart showing the number of participants who were screened and allocated to the comparison group (CG) and AI-intervention group (IG). Exclusions by clinical criteria consist of: (1) new symptoms that demand clearance by another healthcare provider who is not a physical therapist; (2) insufficient improvement or worsening of the condition, suggestive of the need for care escalation.

From a total of 41,993 participants screened for eligibility, 36,186 patients started the study, of which 12,103 participants were in the CG and 24,083 in the IG cohorts (Figure 2).

The defined cohorts were assisted by PTs with a difference in workload (PT-to-patient ratio: IG: 1:129 SD 27 vs. CG 1:57, SD 36) of 2.3× times.

### 3.1. Baseline Characteristics

Both groups exhibited a similar proportion of women (58%, *p* = 0.414) (Table 1). Statistically significant differences between groups were observed in BMI, albeit to a small extent (IG: 39.6%, CG: 37.9%, and *p* < 0.001). On average, the IG cohort was younger, having a lower proportion of patients aged 60 years or older than the CG cohort (16.7% vs. 21.6%, and *p* < 0.001). Racial diversity was observed regardless of the group, with the highest proportion of patients identifying as non-Hispanic whites in both groups (IG: 67.9% and CG: 66.3%). Both groups were composed predominantly of individuals with higher education (considering a bachelor’s or graduate degree: IG: 64.1%; CG: 66.2%). Participants predominantly resided in urban areas, with no significant differences between groups (*p* = 0.077). The IG cohort reported a higher percentage of full-time employment (87.5% vs. 75.7%), while the CG cohort had a greater proportion of part-time workers (16.1% vs. 4.2% in the IG; *p* < 0.001).

Pain level differences between groups were not clinically meaningful [24] (IG: 4.84, SD 1.9 and CG: 4.73, SD 1.9; *p* < 0.001), and no significant differences were observed in terms of anxiety (IG: 8.72, SD 3.94 and CG 8.81, SD 4.06; *p* = 0.083) and depression (IG: 9.52, SD 4.20 and CG: 9.23, SD 4.27; *p* = 0.688).

### 3.2. Outcomes

#### 3.2.1. Clinical Outcomes

Outcome model estimates for the IG and CG groups, adjusted with inverse probability weighting to balance the groups, and respective model fitness are presented in Supplementary Tables S3 and S4, respectively.

Significant improvements in pain were observed in both groups (Supplementary Table S3), as reflected by similar response rates [20]: 64% (95%CI 62–65%) for the IG cohort and 63% (95%CI 61–64%) for the CG cohort (difference between groups: *p* = 0.399; effect size 0.01).

Additionally, significant improvements in the mental health domain were observed similarly in both groups. The anxiety mean change was −4.0 (95%CI −4.2; −3.7) in the IG cohort and −4.1 (95%CI −4.4; −3.9) in the CG cohort, with no significant differences between groups (0.002 (95%CI −0.03; 0.03), *p* = 0.928; effect size: −0.04). Similarly, the depression mean change was −4.0 (95%CI −4.3; −3.7) in the IG cohort and −4.7 (95%CI −5.1; −4.3), in the CG cohort (both *p* < 0.001), regardless of the group (a very small effect size of −0.30; differences between groups: −0.03 (95%CI −0.07; 0.01), *p* = 0.187).

#### 3.2.2. Engagement and Satisfaction

A higher completion rate was observed in the IG cohort (79.9% (N = 19,252)) than in the CG cohort (70.1% (N = 8489), *p* < 0.001). No meaningful differences in the average sessions performed per week were observed between groups, albeit being statistically significant (IG: 2.09, SD 1.03 vs. CG: 2.06, SD 1.16, *p* < 0.001).

There were significantly more reach-outs to patients in the IG cohort (25.11, SD 18.54 vs. 17.33, SD 21.88, in the CG cohort; *p* < 0.001). However, the number of messages initiated by patients remained similar, with the IG cohort sending, on average, 14.6 messages (SD 17.9) compared to 13.9 messages (SD 25.87) in the CG cohort (despite a significance of *p* = 0.018).

High and similar satisfaction with the program was observed in both groups (IG: 8.76 out of 10, SD 1.75, vs. CG: 8.60, SD 1.76, *p* = 0.021), despite the statistical difference.

#### 3.2.3. Adverse Events

Intervention-related adverse events were rare and were evenly distributed across the groups, with the IG cohort reporting 140 (0.58%) and the CG cohort 83 (0.69%) (*p* = 0.231), detailed description in Supplementary Table S5. Condition-related (e.g., surgery) and condition/intervention-unrelated adverse events (e.g., respiratory infections, allergic reactions, falls outside intervention, cancer onset, etc.) accounted for the majority of the total adverse events (89.8%; N = 1963/2186).

## 4. Discussion

### 4.1. Main Findings

In this study, patient engagement, satisfaction, clinical outcomes, and adverse events were evaluated before and after a 2.3-fold increase in PT-to-patient ratio, supported by an AI tool integrated in the PT clinical portal. Similar clinical outcomes (pain, anxiety, and depression) were observed across both groups (denoted by very small effect sizes: −0.30–0.01, *p* = 0.187–0.928).

Engagement was not negatively affected, with both groups presenting a similar frequency of sessions per week (IG: 2.09, SD 1.03 vs. CG: 2.06, SD 1.16, *p* < 0.001) and with the IG cohort reporting higher completion rates (79.9% (N = 19,252) vs. 70.1% (N = 8489), *p* < 0.001). Reach-outs initiated by the PT were more frequent in the IG (25.1 SD 18.5 vs. 17.3 SD 21.9, *p* < 0.001), while communication initiated by the members remained stable between groups (IG: 14.6, SD 17.9 vs. CG: 13.9, SD 25.9, *p* = 0.018). Satisfaction with the program was consistently high for both groups (IG: 8.76 out of 10, SD 1.75 vs. CG: 8.60, SD 1.76; *p* = 0.021). Crucially, the rate of adverse events remained consistently low across both groups.

All the above seem to suggest that care scalability is possible through the integration of AI tools in the workflow, without compromising clinical outcomes or program safety.

### 4.2. Comparison with Previous Studies

System sustainability promotion [30] and improvement in care delivery efficiency [8,10] have been fostered by AI transformational force in several healthcare sectors [31,32,33,34,35]. However, the application of AI in MSK care is still in its early stages.

Safety, interpretability, and transparency are key factors when developing AI tools. Many guidelines and research are now available to guide AI tool design following responsible AI principles [18,36,37]. Herein, we used a human-in-the-loop approach [38] where the final decision always rests with the healthcare provider. Clinicians can then validate AI-generated recommendations, correct errors, and provide additional insights based on their experience and expertise. Additionally, interpretability and transparency can be fostered by a more complete interface where the rationale supporting a given recommendation is provided [36,39], as was the case with the described AI tool.

To our knowledge, this is the first study assessing the outcomes after scaling an MSK management program leveraging the implementation of an AI-based system to streamline PT workflow. An encouraging finding of the present study was that neither engagement nor patient satisfaction was negatively impacted following scaling. This contrasts with the concerns expressed by both clinicians and patients that remote interventions, especially those operating at scale, might lead to reduced patient accountability, motivation, and adherence [40,41]. Completion rate was increased in the IG, reinforcing the lack of negative outcome from the scale context. The higher number of PT reach-outs in the IG may have contributed to increased engagement. This could be associated with streamlined workflows and the reduced burden of administrative tasks, diverting more time to patient contact. Additionally, a higher number of dropouts and unrelated medical exclusions were reported in the CG, which was possibly associated with the particular timeframe of the cohort in question (including the COVID-19 pandemic), which may have also contributed to a lower completion rate.

Importantly, clinical outcomes remained fairly stable between cohorts, as testified by the similar response rate for pain (64% vs. 63%, *p* = 0.399), and also in the mental health domain (anxiety change difference: 0.002 (95%CI −0.03; 0.03), *p* = 0.928, effect size: −0.04, and depression change difference: −0.03 (95%CI −0.07; 0.01), *p* = 0.187, effect size: −0.30). The feedback loop between pain intensity and mental health issues is well known [42], as is the impact of a proper therapeutic alliance to address these intricacies and to effectively support condition management [43]. At the same time, and despite the increase in the PT-to-patient ratio, the patients’ ability to communicate with their assigned PT was not affected, as evidenced by the similar number of communications initiated by the patients themselves in both groups (IG: 14.6 SD 17.9 vs. CG: 13.9 SD 25.9). As explored in other digital interventions, the ability to reach the clinician when needed has been reported as pivotal in initiating and maintaining a trust relationship that fosters better outcomes [40,44,45].

The stability of clinical outcomes between groups seems to advocate for care scalability and the use of technology to support PTs’ clinical work, which follows the trend reported previously for AI tools developed for other healthcare professionals [10,46]. Crucially, the adverse event rate remained low and consistent despite the increase in the PT-to-patient ratio, underscoring the priority given to safety throughout the design, development, and implementation of this AI tool into the PTs’ workflow. The adverse event rate herein reported was lower than that described in in-person interventions [47,48]. Specifically, in real-world digital settings, adverse events are scarcely reported, with a large retrospective study also reporting a low adverse event rate following the use of a self-supporting app [49]. This scarcity could be associated with the lack of the systematic collection and reporting of adverse events, particularly when considering that most digital interventions are self-managed without the support of a healthcare professional [50].

Overall, the findings of this study support the feasibility and safety of scaling remote care, utilizing AI-based tools to expand the outreach of healthcare professionals. These results align with a recent study that surveyed the perspectives of physical therapists regarding AI in healthcare, wherein 78.2% of participants agreed or strongly agreed that implementing AI in healthcare could increase work productivity [51,52].

### 4.3. Strengths and Limitations

The primary strength of the current study lies in its novelty, as it directly evaluates the scalability of MSK care that is supported by an AI-based system, using a large and diverse sample from a real-world context. Importantly, with the rise of AI tool development and adoption, concerns and awareness have been raised about the risks of poor AI conceptualization/design [53]. Therefore, the WHO guidelines [18] stress the importance of preserving the epistemic authority of healthcare providers in medical decision-making over the influence of automated (and sometimes biased and/or black-boxed) AI systems. Following WHO best practice, the AI tool herein described adopted a human-in-the-loop framework, not only to develop the tool itself but also to guarantee that all final decisions were made after critical reasoning and assessment by PTs. Moreover, the continuous internal quality check of the AI tool’s performance against PT manual decisions safeguards against the possibility of poor recommendations.

However, there are limitations to consider. This non-randomized, controlled before-and-after study lacks randomization between groups, which was mitigated through the application of inverse probability weighting during statistical analysis. Additionally, the absence of an additional control group undergoing PT-to-patient ratio scaling without the implementation of an AI tool could have helped mitigate the influence of unaccounted confounding variables. However, ethical considerations prevented the pursuit of this strategy as safety and effectiveness concerns outweighed the potential additional insights that it could have provided. Consequently, there remains the possibility of other confounding variables that are not addressed in the study influencing outcomes, such as the cohort’s timeframe or the potential use of complementary treatments. While the study addresses a large sample size, the study population included the beneficiaries of employer health plans; therefore, further investigation in other cohorts should be carried out. Finally, future research should consider an evaluation of workflow time savings and qualitative analysis of the providers’ perceptions of the tool for further workflow sustainability and increased workforce wellbeing.

## 5. Conclusions

This is the first study demonstrating that similar clinical outcomes (pain, anxiety, and depression) can be attained after an increase in the PT-to-patient ratio that is supported by the implementation of an AI-based system to streamline PT workflow. Moreover, patient engagement was not impacted, as evidenced by higher completion rates and a consistent level of satisfaction with the program. The stable adverse event rate across groups further supports the safety of scaling care through AI. The study advances current knowledge by underscoring the potential of AI technology in MSK management, particularly to enhance PT workflows. Future research should aim to clarify and quantify the gains in workflow efficiency and the associated cost-effectiveness of such approaches, as well as the perceptions of providers regarding the impact of such tools on their work.

## Figures and Tables

**Table 1:** Baseline characteristics for the AI-intervention group (IG) and comparison group (CG), following an intention-to-treat analysis. Filtered cases correspond to participants who presented with clinically relevant baseline scores (≥5) in terms of mental health variables.

	IG (N = 24,083)	CG (N = 12,103)	*p*-Value
Age (years), mean (SD)	48.5 (11.6)	50.0 (11.7)	<0.001
Age categories (years), N (%):			
<25	342 (1.4)	72 (0.6)	<0.001
25–40	6099 (25.3)	2795 (23.1)	
41–60	13,615 (56.5)	6623 (54.7)	
>60	4027 (16.7)	2613 (21.6)	
Gender, N (%) ^a^:			
Woman	14,126 (58.7)	6984 (57.9)	0.414
Man	9849 (41.0)	5044 (41.8)	
Non-binary	67 (0.3)	35 (0.3)	
Other	4 (0.0)	1 (0.0)	
BMI (kg/m^2^), mean (SD) ^b^	29.7 (7.0)	29.3 (6.7)	<0.001
BMI categories (kg/m^2^), N (%) ^b^:			
Underweight (<18.5)	199 (0.8)	102 (0.8)	0.001
Normal (18.5–25)	6223 (25.9)	3349 (27.7)	
Overweight (≥25–30)	8100 (33.7)	4051 (33.5)	
Obese (≥30)	9528 (39.6)	4584 (37.9)	
Race/ethnicity, N (%) ^c^:			
Asian	2311 (9.8)	928 (10.2)	0.017
Black	2297 (9.8)	982 (10.8)	
Hispanic	2268 (9.6)	890 (9.8)	
Non-Hispanic White	15,991(67.9)	6013 (66.3)	
Other	690 (2.9)	250 (2.8)	
Education level, N (%) ^d^:			
Less than high school diploma	219 (0.9)	74 (0.7)	0.001
High school diploma	2172 (9.1)	823 (8.1)	
Some college	6133 (25.8)	2528 (25.0)	
Bachelor’s degree	9397 (39.5)	4162 (41.2)	
Graduate degree	5846 (24.6)	2523 (25.0)	
Geographic location, N (%) ^e^:			
Urban	21,321 (88.8)	10,767 (89.4)	0.077
Rural	2693 (11.2)	1276 (10.6)	
Employment status, N (%) ^f^:			
Full-time job	20,807 (87.5)	8987 (75.7)	<0.001
Part-time job	997 (4.2)	1908 (16.1)	
Retired	991 (4.2)	608 (5.1)	
Not employed	990 (4.2)	376 (3.2)	
**Clinical data, mean (SD)**			
Analgesic intake, N (%)	5580 (23.2)	2850 (23.6)	0.423
Symptomatic anatomical area:			
Ankle	1395 (5.8)	482 (4.0)	<0.001
Elbow	523 (2.2)	262 (2.2)	
Hip	2592 (10.8)	1267 (10.5)	
Knee	3633 (15.1)	1832 (15.1)	
Low back	8589 (35.7)	4590 (37.9)	
Neck	2601 (10.8)	1304 (10.8)	
Shoulder	3730 (15.5)	1928 (15.9)	
Wrist	1020 (4.2)	438 (3.6)	
Pain intensity ^c^, mean (SD)	4.73 (1.9)	4.84 (1.9)	<0.001
Mental health ≥ 5, mean (SD):			
GAD-7 ^g^	8.72 (3.94)	8.81 (4.06)	0.083
PHQ-5 ^h^	9.52 (4.20)	9.23 (4.27)	0.688

Data represent the mean ± standard deviation or the number of patients and the percentage of the total where listed. Missing values or prefer not to answer: ^a^ 76, ^b^ 50, ^c^ 3566, ^d^ 2309, ^e^ 129, ^f^ 522. ^g^ GAD-7 ≥ 5: IG = 3645 (30.2%) and CG = 8347 (34.7%); ^h^ PHQ-9 ≥ 5: IG = 2803 (23.2%) and CG = 6192 (25.7%). Abbreviations: BMI, body mass index; GAD-7 generalized anxiety disorder 7-item scale; PHQ-9 patient health 9-item questionnaire.

## Data Availability

The data presented in this study are available on request from the corresponding author. The data are not publicly available due to privacy restrictions.

## Supplementary Materials

The following supporting information can be downloaded at: https://www.mdpi.com/article/10.3390/jcm13154366/s1, Figure S1: Participants performing exercise sessions of their digital care pro-gram for (a) wrist/hand, and (b) low back conditions; Table S1. Description of the exercise prescription; Table S2. Input variables feeding the AI tool and example of the AI model output to the physical therapist; Table S3: Model estimates of clinical outcomes for each group, following an intent-to-treat analysis; Table S4: Latent growth curve model fit; Table S5: Adverse events related to the intervention registered for both I-intervention group (IG) and control group (CG).
