# Supplementary material for: Applying AI to Safely and Effectively Scale Care to Address Chronic MSK Conditions
Source: J Clin Med. 2024 Jul 26;13(15):4366. doi: 10.3390/jcm13154366 (PMC11312972; doi:10.3390/jcm13154366)
Supplement: Supplementary file 1 [file jcm-13-04366-s001.zip › jcm-3012587-supplementary.pdf]

Supplementary Materials

Applying AI to safely and effectively scale care to address chronic MSK conditions

Anabela C. Areias; Dora Janela; Robert G. Moulder; Maria Molinos; Virgílio Bento; Carolina Moreira; Vijay Yanamadala; Fernando Dias Correia; Fabíola Costa

Supplementary Figure S1. Participants performing exercise sessions of their digital care program for a) wrist/hand, and b) low back conditions.

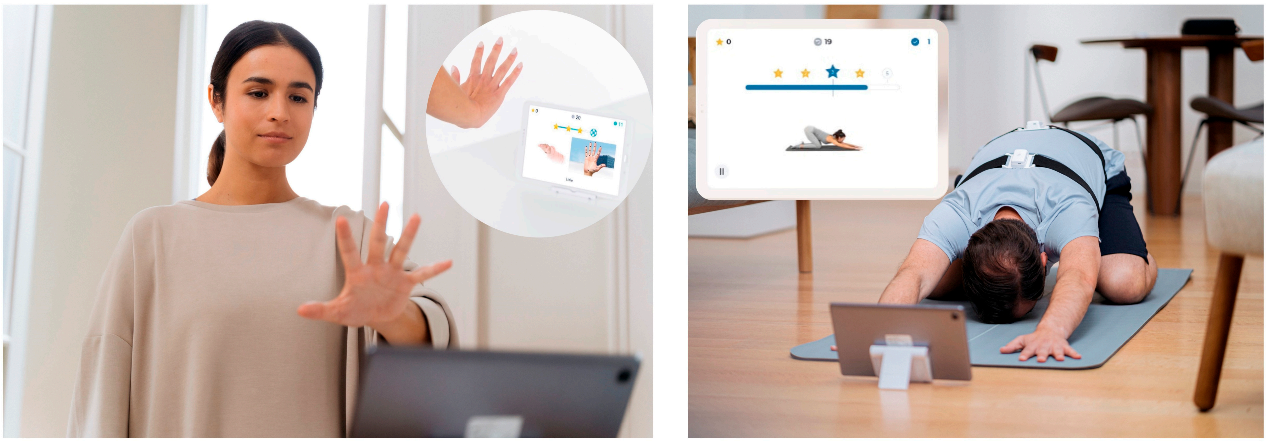

Supplementary Table S1. Description of the exercise prescription.

Phases and Main goals	Intervention description
**Initial Phase**<br><br>Goals:<br>● Decrease symptoms and inflammatory signs<br>● Gradual exposure to avoided movements<br>● Improve range of motion (passive and active)<br>● Improve muscular strength and endurance<br>● Improve motor control and stability<br>● Provide knowledge on the condition, contributing factors and the role of exercise	Aim for at least 1-2 sets of:<br>● Articular mobility exercises<br>● Strengthening exercises<br>● Active and passive stretching exercises
**Intermediate Phase**<br><br>Goals:<br>● Improve range of motion<br>● Increase global muscular and aerobic capacity<br>● Increase time of exposure to painful movements<br>● Gradual exposure to daily activities that elicit symptoms	Increase repetitions and sets or add external load (bands and/or free weights) of the prescribed exercises; Add more demanding exercises<br><br>Progressively increase average session time<br><br>Increase exercise complexity and aim for at least 2-3 sets of:<br>● Articular mobility exercises (e.g. standing trunk movements)

	● Strengthening exercises (e.g. isometric wall squat; plank)<br>● Active and passive stretching exercises (standing anterior thigh stretch)
**Late Phase**<br><br>Goals:<br>● Functional restoration of range of motion<br>● Return to daily activities<br>● Increase time of exposure to daily activities that elicit symptoms<br>● Foster independence	Add or increase external load of prescribed exercises (bands and/or free weights)<br><br>Increase complexity and add single leg stance exercises:<br>● Articular mobility exercises (e.g. single leg knee to chest)<br>● Strengthening exercises (e.g. plank with trunk movement or with hand movement; lunges)<br>● Active and controlled stretching exercises

Notes:
In addition to anamnesis, physical assessment comprised the evaluation of movement patterns, addressing compensations/difficulties during movements executions, and active and passive range of motion in uni-joint and multi-joint movements (measured by the motion tracking technology).
The exercise prescription was tailored according to the initial assessment and individual patient progress during the study.
The following parameters of the exercise prescription were adjusted by the physical therapist according to the patient's evolution: range of motion, number of exercises, number of sets and repetitions and the type of exercise. Alongside assessment and communication between patients and PT, the performance (namely the range of motion, execution, movement compensations and skipped exercises) and the level of pain and fatigue during exercises reported by the patient were taken in consideration for the intervention decision making.

Supplementary Table S2. Input variables feeding the AI tool and example of the AI model output to the physical therapist.

**Examples of the functioning of the AI-tool**	
**Input data**	Total sessions completed by the patient;<br>Average and maximal performance during exercise sessions[a];<br>Pain and fatigue level reported during sessions;<br>Exercises skipped during sessions;
**Example of recommendation to PT**	Recommendation: “Member is recommended to progress to the next phase”;<br><br>Recommendation reasons:<br>High performance in the last 3 sessions<br>Low Pain during exercising in the last sessions<br>No skipped exercises in the last sessions

[a]Corresponds to the sum of correct movements divided by the sum of total movements (independently if correct or incorrect) for each session.

Supplementary Table S3. Model estimates of clinical outcomes for each group following an intent-to-treat analysis.

Outcome mean (95%CI)	Intercept				Slope				Curve				Change Estimates	
	IG	CG	*Difference*	*P*	IG	CG	*Difference*	*P*	IG	CG	*Difference*	*P*	*IG*	*CG*
Pain	4.73 (4.70;4.75)	4.84 (4.80;4.87)	-0.11 (-0.15;-0.07)	<.001	-0.17 (-0.18;-0.16)	-0.18 (-0.19;-0.17)	0.01 (0.00;0.02)	.012	0 (0;0)	0 (0;0)	0 (0;0)	.064	-1.9 (-1.9;-1.8)	-1.9 (-2.0;-1.8)
GAD-7 ≥5	8.68 (8.59;8.76)	8.77 (8.64;8.91)	-0.10 (-0.25;0.06)	.228	-0.39 (-0.42;-0.38)	-0.40 (-0.42;-0.37)	0.001 (-0.03;0.03)	.928	0.01 (0.01;0.01)	0.01 (0.01;0.01)	0 (0;0)	.774	-4.0 (-4.2;-3.7)	-4.1 (-4.4;-3.9)
PHQ-5 ≥5	9.46 (9.36;9.57)	9.23 (9.07;9.39)	0.23 (0.04;0.42)	.019	-0.48 (-0.50;-0.45)	-0.45 (-0.48;-0.41)	-0.03 (-0.07;0.01)	.187	0.01 (0.01;0.01)	0.01 (0.01;0.01)	0 (0;0)	.017	-4.0 (-4.3;-3.7)	-4.7 (-5.1;-4.3)

Abbreviations: CG, Comparison group; GAD-7 Generalized Anxiety Disorder 7-item scale; IG: Intervention group; PHQ-9 Patient Health 9-item questionnaire.

Supplementary Table S4. Latent growth curve model fit.

Outcome	Chi-sq (df)	*P-value*	CFI	RMSEA	SRMR
Pain	2084.51 (2)	< .001	**0.93**	**0.040**	0.075
GAD-7 ≥5	205.79 (2)	< .001	**0.96**	**0.056**	**0.043**
PHQ-5 ≥5	134.67 (2)	< .001	**0.96**	**0.052**	**0.039**

Note: If a significant chi-square is found for a model, then comparative fit index values (CFI) > .9, or root mean square error of approximation values (RMSEA) < .08, or standardized root mean squared residual values (SRMR) <.05 signify models with acceptable fit. Parameters denoting acceptable fit are presented in bold.

Supplementary Table S5. Adverse events related to intervention registered for both intervention group (IG) and control group (CG).

	IG (N=24,083)	CG (N=12,103)	P-value
Intervention-related	140	83	0.231
Increased Pain	77	41	
Aggravated condition	8	6	
New Pain	3	3	
Injury (e.g. dislocation, injured muscle)	8	5	
Muscle Issues	18	7	
Inflammation	0	1	
Swelling	5	8	
Fall	3	5	
Unpleasant sensation (e.g. nausea, dizziness)	12	5	
Straps skin reaction	3	1	
Other adverse events (e.g. migraines, vertigo)	3	1	
